# Fabrication of piezoelectric poly(l-lactic acid)/BaTiO_3_ fibre by the melt-spinning process

**DOI:** 10.1038/s41598-020-73261-3

**Published:** 2020-10-01

**Authors:** Hyun Ju Oh, Do-Kun Kim, Young Chan Choi, Seung-Ju Lim, Jae Bum Jeong, Jae Hoon Ko, Wan-Gyu Hahm, Sang-Woo Kim, Yongju Lee, Hyeok Kim, Byeong Jin Yeang

**Affiliations:** 1 Advanced Textile R&D Department, Korea Institute of Industrial Technology, Ansan, Republic of Korea; 2 Department of Chemical Engineering, Hanyang University ERICA, Ansan, Republic of Korea; 3 Department of Advanced Materials Engineering for Information & Electronics, Kyung Hee University, Yongin, Republic of Korea; 4 Nano Materials & Nano Technology Center, Korea Institute of Ceramic Engineering and Technology, Jinju, Republic of Korea; 5 School of Advanced Materials Science and Engineering, Sungkyunkwan University (SKKU), Suwon, 16419 Republic of Korea; 6 School of Electrical and Computer Engineering, University of Seoul, Seoul, Republic of Korea

**Keywords:** Sensors and biosensors, Mechanical engineering, Materials science

## Abstract

Poly(l-lactic acid) (PLLA) based piezoelectric polymers are gradually becoming the substitute for the conventional piezoelectric ceramic and polymeric materials due to their low cost and biodegradable, non-toxic, piezoelectric and non-pyroelectric nature. To improve the piezoelectric properties of melt-spun poly(l-lactic acid) (PLLA)/BaTiO_3_, we optimized the post-processing conditions to increase the proportion of the β crystalline phase. The α → β phase transition behaviour was determined by two-dimensional wide-angle x-ray diffraction and differential scanning calorimetry. The piezoelectric properties of PLLA/BaTiO_3_ fibres were characterised in their yarn and textile form through a tapping method. From these results, we confirmed that the crystalline phase transition of PLLA/BaTiO_3_ fibres was significantly enhanced under the optimised post-processing conditions at a draw ratio of 3 and temperature of 120 °C during the melt-spinning process. The results indicated that PLLA/BaTiO_3_ fibres could be a one of the material for organic-based piezoelectric sensors for application in textile-based wearable piezoelectric devices.

## Introduction

Owing to their stretchability, flexibility, and easy fabrication, fibrous piezoelectric materials have attracted a great deal of attention for the manufacture of wearable electronic devices that can detect motion as well as physiological and behavioural signals. Fibrous piezoelectric sensors can be incorporated into clothing and accessories, such as watches, eyeglasses, and bracelets, or even mounted directly on the body^1–5^. As a person moves, the motion causes the piezoelectric fibrous sensor to stretch, twist, bend, or shear; this mechanical deformation is converted into an electrical signal that can be used to monitor human physiological conditions. Various approaches have been used to produce polymer-based fibrous sensors. These include wet-spinning, electrospinning, melt-spinning, film-drawing, and the incorporation of fibre composites^6–11^. However, to manufacture polymer-based piezoelectric fibrous sensors for practical applications, an economical processes suitable for mass production is needed. In this respect, the melt-spinning process may be the most promising for facile and low-cost mass production.

Poly(l-lactic acid) (PLLA)-based piezoelectric polymers are gradually replacing conventional ceramic and polymeric piezoelectric materials, because they are economical, biodegradable, non-toxic, and non-pyroelectric. The thermal, mechanical, and electrical properties of PLLA are significantly dependent on the crystalline phases it contains^12–17^. PLLA can contain three different crystalline phases—α, β, and γ—depending on the processing conditions. The most common and stable polymorph is the α-phase, which exhibits a 10_3_ helical chain conformation and a pseudo-orthorhombic unit cell. It can be obtained easily via melting or solution-phase crystallization methods. The β-phase, which has a left-handed 3_1_ helical conformation and an orthorhombic or triclinic unit cell, can be produced by controlling the post-process drawing and annealing conditions. The γ-phase has recently been obtained through epitaxial growth of PLLA on a hexamethylbenzene (HMB) substrate.

The C=O dipoles of α-PLLA are generally oriented in all directions (360°) along the main chain of the helical structure, resulting in a zero net-dipole moment. α-PLLA can be transformed to β-PLLA by a stretching or drawing processes. The C=O dipoles of β-PLLA are aligned along the backbone chain, so the total dipole moment is zero due to its helical structure. The shear piezoelectricity of PLLA, or its piezoelectric-like behaviour^18,19^, is of considerable interest to researchers. Shear piezoelectricity (*d*_14_) is induced along the primary axis of PLLA upon the application of shear stress, as the C=O dipoles in the helical backbone rotate, causing electrical polarization. In contrast, the β-crystalline phase of polyvinylidene fluoride (PVDF) can have a large net polarity, because it has a linear molecular structure. PLLA-based piezoelectric materials generally have lower piezo-responses than PVDF or ceramic materials based on lead zirconate titanate (PZT). However, the proportion of β-phase can be increased by adding ferroelectric particles, such as barium titanate (BaTiO_3_)^20^, iron oxide (Fe_2_O_3_)^21^, and zinc oxide (ZnO)^22^ and this improves the electrical properties parameters. It can also be increased by adjusting post-processing parameters. Xu et al.^23^ and Takahashi et al.^24^ investigated the effects of drawing stress, drawing temperature, and draw ratio on the crystalline phase transition in PLLA films containing highly oriented α-crystals. Lee et al.^19^ reported that the piezoelectricity of PLLA nanofibers was enhanced by the application of the electric field in electrospinning processes. They also found that the orientation of PLLA nanoweb stacking and the thickness of electrospun PLLA nanofiber webs could be controlled to increase shear piezoelectricity. However, these materials were in unwoven film form and were thus limited with regard to fabricating wearable sensor materials.

In this work, we investigated the effects of adjusting the draw ratio and temperature of the melt-spinning process on the piezoelectric properties of PLLA/BaTiO_3_ fibres. The crystal transitions and orientations of the PLLA/BaTiO_3_ fibres obtained under the various post-processing conditions were examined by differential scanning calorimetry (DSC) and two-dimensional wide-angle X-ray diffraction (2D-WAXD). The tensile strength of the PLLA/BaTiO_3_ fibres was measured with a universal testing machine. The piezoelectricity of the PLLA/BaTiO_3_ fibres and piezoelectric textile were determined by monitoring the output voltage and current with a force tapping method.

## Results

**Figure 1:**
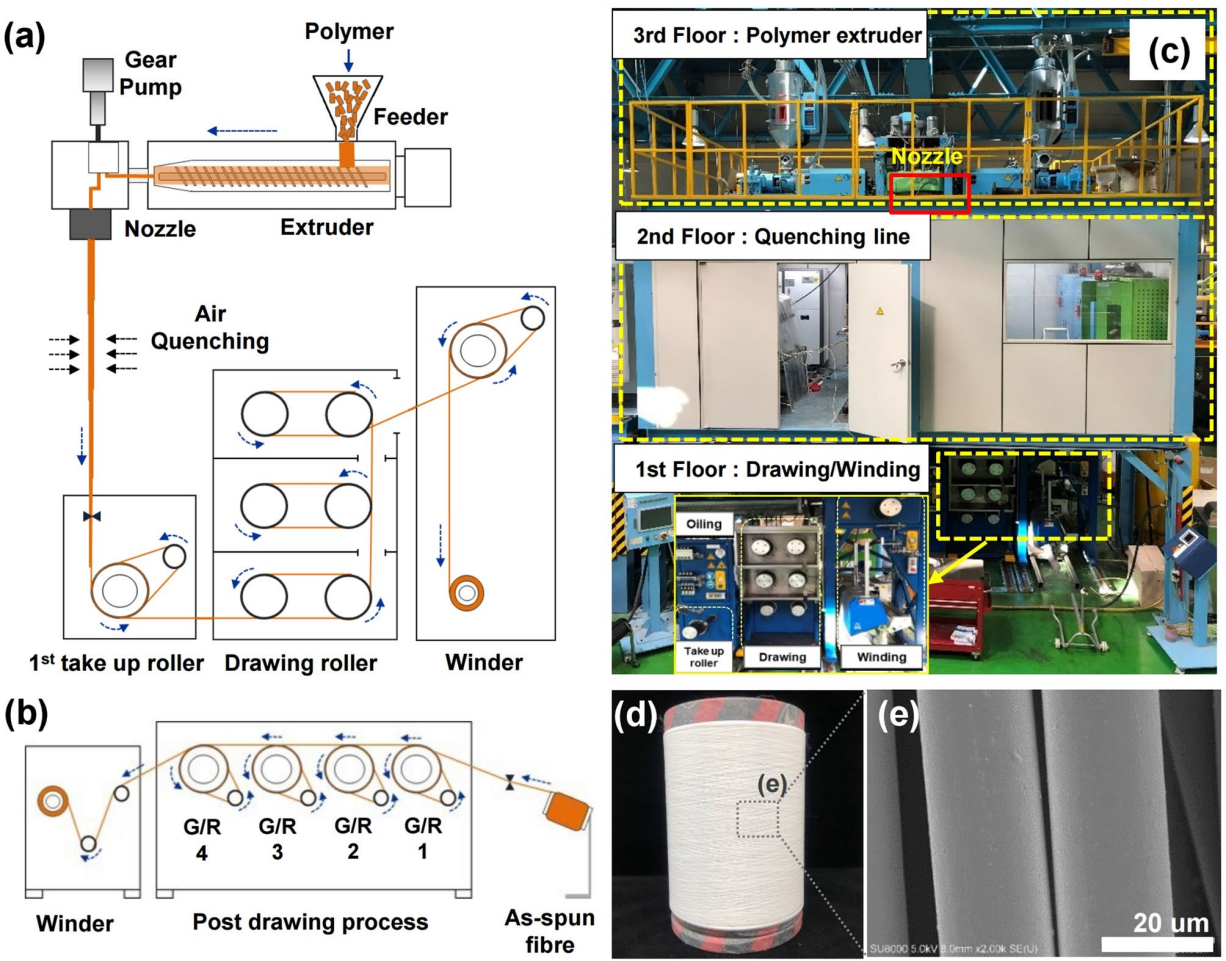
Schematic diagram of (**a**) the pilot-scale melt-spinning apparatus, (**b**) post-drawing process, (**c**) photographs of our pilot-scale melt-spinning apparatus, (**d**) obtained as-spun PLLA fibre reinforced with BaTiO_3_ particles by the pilot-scale melt-spinning, and (**e**) FE-SEM image of the as-spun PLLA/BaTiO_3_ fibres.

**Figure 2:**
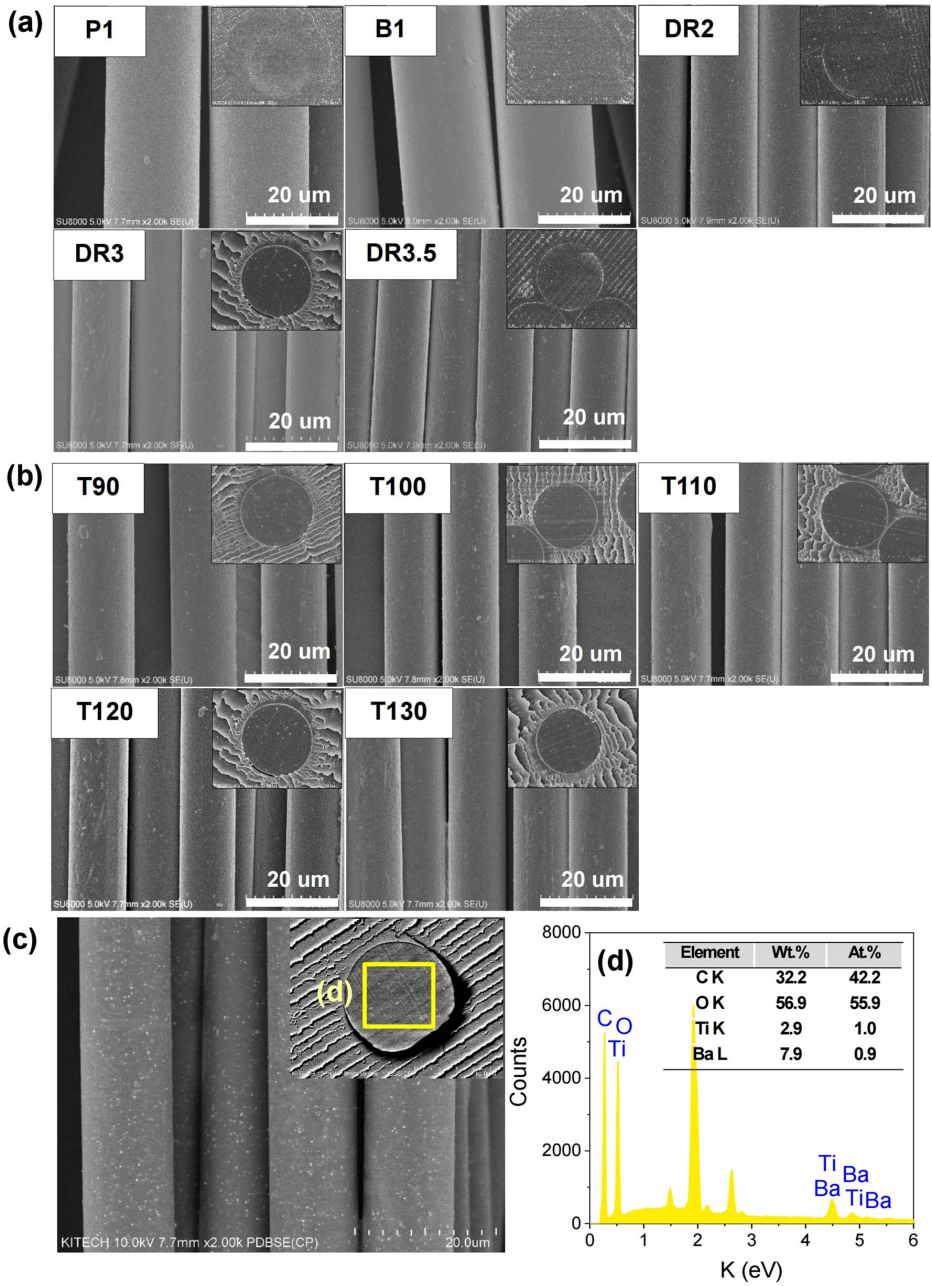
FE-SEM images of the PLLA/BaTiO_3_ fibres. Surface and cross-sectional images of the as-spun fibres and fibres obtained with (**a**) various draw ratios (DR) and (**b**) temperatures (T, °C). (**c**) Dispersion of BaTiO_3_ particles in the PLLA fibre (T120) using BSE image mode and (**d**) EDS element spectra.

A schematic diagram and photograph of a pilot melt-spinning apparatus are shown in Fig. 1a–c. The post-drawing process is illustrated schematically in Fig. 1b. To increase the proportion of crystalline β-phase in the as-spun PLLA fibres (Fig. 1d,e), the post-process draw ratio and the temperature of the off-line drawing system were controlled. The surface field emission-scanning electron microscopy (FE-SEM) images of the as-spun PLLA/BaTiO_3_ fibres are shown in Fig. 1e. The drawing conditions of each samples are presented in Table 1. The Surface and cross-sectional FE-SEM images of the pristine PLLA and PLLA/BaTiO_3_ fibres prepared at various draw ratios and temperatures are shown in Fig. 2. The diameters obtained with various draw ratios and temperatures are summarized in Table 1. Figure 2a shows the decrease in the diameter of the PLLA/BaTiO_3_ fibres as the draw ratio increased. Compared to the undrawn as-spun fibre, a diameter of 12.6 µm is obtained at a maximum draw ratio of 3.5 (DR3.5). For the temperature variations at a draw ratio of 3, there is no change observed in the diameter with a similar distribution for PLLA/BaTiO_3_ fibres in the range of 13.8 to 13.0 µm (Fig. 2b). The dispersion of the BaTiO_3_ nanoparticles in the PLLA fibres (T120) was characterised by the back-scattered electron (BSE) imaging mode. BaTiO_3_ nanoparticles were relatively well-dispersed in the PLLA fibres as shown in Fig. 2c. The results of the energy-dispersive spectroscopy (EDS) analysis of the PLLA/BaTiO_3_ fibres are shown in Fig. 2d and the inset table.

**Table 1:** Melt-spun PLLA/BaTiO_3_ fibre labels with drawing conditions, fibre diameters.

Label	BaTiO_3_, wt%	Draw ratio	Draw temperature, (°C)	Fibre diameter, (µm) ± STDV
P1	0	1	–	22.4 ± 0.80
B1	3	1	–	24.1 ± 0.77
DR2	3	2	120	15.7 ± 0.60
DR3	3	3	120	13.3 ± 0.47
DR3.5	3	3.5	120	12.6 ± 0.58
T90	3	3	90	13.9 ± 0.54
T100	3	3	100	13.2 ± 0.82
T110	3	3	110	12.9 ± 0.54
T120	3	3	120	13.3 ± 0.47
T130	3	3	130	13.0 ± 0.44

**Figure 3:**
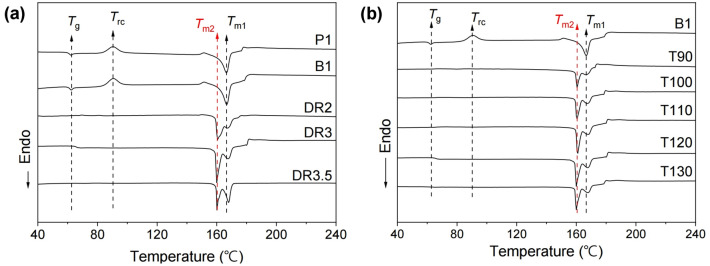
DSC thermograms of PLLA fibres reinforced with BaTiO_3_ nanoparticles obtained with various (**a**) draw ratios (DR) and (**b**) temperatures (T, °C).

The DSC thermograms of the PLLA/BaTiO_3_ fibres treated with various draw ratios and temperatures are shown in Fig. 3. The glass transition temperature (*T*_g_) and recrystallization temperature (*T*_rc_) of the as-spun P1 and B1 fibres were 62.4 and 90.6 °C as shown in Fig. 3a, respectively. The melting temperature (*T*_m1_) was also approximately 166.7 °C and it was represented by a single melting peak. Interestingly, the glass transition and recrystallization peaks of the PLLA/BaTiO_3_ fibres completely disappeared after the drawing process, and a second melting peak appeared in the thermogram. This second melting temperature (*T*_m2_) was approximately 7 °C lower than the first (*T*_m1_). The area of each endothermal peak at *T*_m2_, which reflects the melting enthalpy, was also larger than that of the peak at *T*_m1_. It has been shown that the melting point of β- PLLA is 10 °C lower than that of α-PLLA due to the lower thermal stability and highly oriented structure of the β-phase^25^. Therefore, the emergence of the second melting peak was likely due to the α → β crystalline phase transition of PLLA. Notably, the melting enthalpy at *T*_m2_ gradually increased and shifted to a higher temperature with each DR increment; the enthalpy at *T*_m1_ simultaneously decreased. However, at a DR3.5, the endothermal peak at *T*_m2_ was decreased, and the peak at *T*_m1_ increased. When the draw stress in the fibres increases, the degree of crystal transition can be improved by lamellae separation. At a high draw ratio, the structural changes such fragmentation and reorganization occurs, and it also induces the lamellae deformation and breakages^26^. In our post process, the fibre breakage occurred at a draw ratio of 4, and DR3.5 was a draw ratio before breakage. It is assumed that the higher peak shift at DR3.5 is caused by the lamellae deformation and initial stage of breakage above a higher draw ratio. The DSC thermograms of the PLLA/BaTiO_3_ fibres as a function of drawing temperature are shown in Fig. 3b. Neither a *T*_g_ nor a *T*_rc_ peak was observed in the thermogram of the fibres treated at a draw temperature of 90 °C, but a *T*_m2_ peak was visible. The peak at *T*_m2_ was shifted to a lower temperature, and the area of the *T*_m2_ peak gradually increased with increasing drawing temperature. In addition, the peak at *T*_m1_ shifted to a higher temperature with increasing drawing temperature. The area of the peak at *T*_m2_ reached a maximum at drawing temperatures approaching 120 °C. However, the crystal transition and crystallinity was decreased at higher temperature (T130).This results is related to a decrease in tensile stress applied to the fibres as temperature increases^27^.

**Figure 4:**
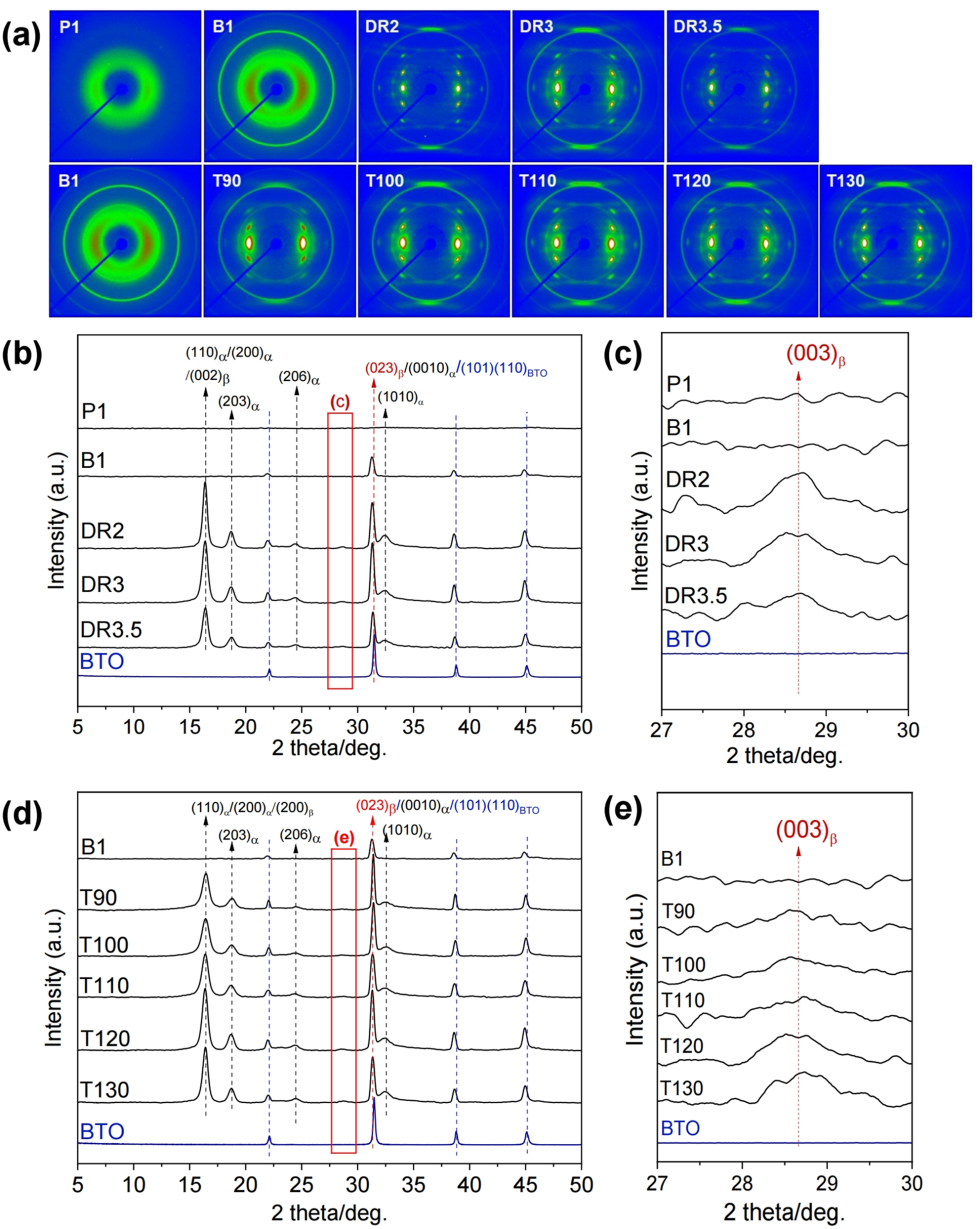
(**a**) 2D-WAXD patterns of PLLA/BaTiO_3_ fibres obtained at various draw ratios (DR) and temperatures (T, °C). XRD patterns of PLLA/BaTiO_3_ fibres obtained with various (**b**,**c**) draw ratios (DR) and (**d**,**e**) temperatures (T, °C).

The 2D-WAXD patterns of the PLLA/BaTiO_3_ fibres are shown in Fig. 4a. An amorphous halo was observed in the as-spun P1 specimens obtained at a take-up velocity of 1 km/min. The as-spun B1 was also showed the amorphous states but it was slightly developed compared to that of pristine one. Crystal formation was induced by the drawing process due to the molecular orientation in the amorphous and meso-phases of the PLLA/BaTiO_3_ fibres during melt-spinning. The XRD patterns collected from (2θ) 5° to 50° are shown in Fig. 4b–e. The diffraction pattern of P1 confirmed the presence of the amorphous phase. In case of B1, the amorphous phase with a sharp peak from the (110) plane of BaTiO_3_ (BTO) nanoparticles was observed. The (110)_α_/(200)_α_/(200)_β_, (203)_α_, (003)_β_, (023)_β_/(0010)_α_/(101)(110)_BTO_ and (1010)_α_ peaks at 16.4°, 18.8°, 28.8°, 31.4° and 32.5°, were assigned to the α- and β-crystalline phases of PLLA and BTO nanoparticles formed during the drawing process^13,24^. As the draw ratio was increased, the intensity of the crystal plane peak at 16.4° gradually increased, and a strong diffraction peak at 31.4° from the BaTiO_3_ nanoparticles overlapping the peaks of the α- and β-crystalline phases increased significantly. However, these peak were diminished at a higher draw ratio (DR3.5). In Fig. 4c, the weak diffraction peak of (003)_β_ is described as indicated by a similar tendency. With respect to the drawing temperature, the intensity of peaks assigned to the α- and β-crystalline phases in PLLA/BaTiO_3_ gradually increased as the drawing temperature increased. The peak from the (003) plane of the β-crystalline phase appeared in the pattern of the sample drawn at 90 °C and gradually increased until the draw temperature reached 130 °C, while the strong reflections of the (110)/(200) planes of the α-crystalline phase decreased at drawing temperatures above 130 °C.

**Figure 5:**
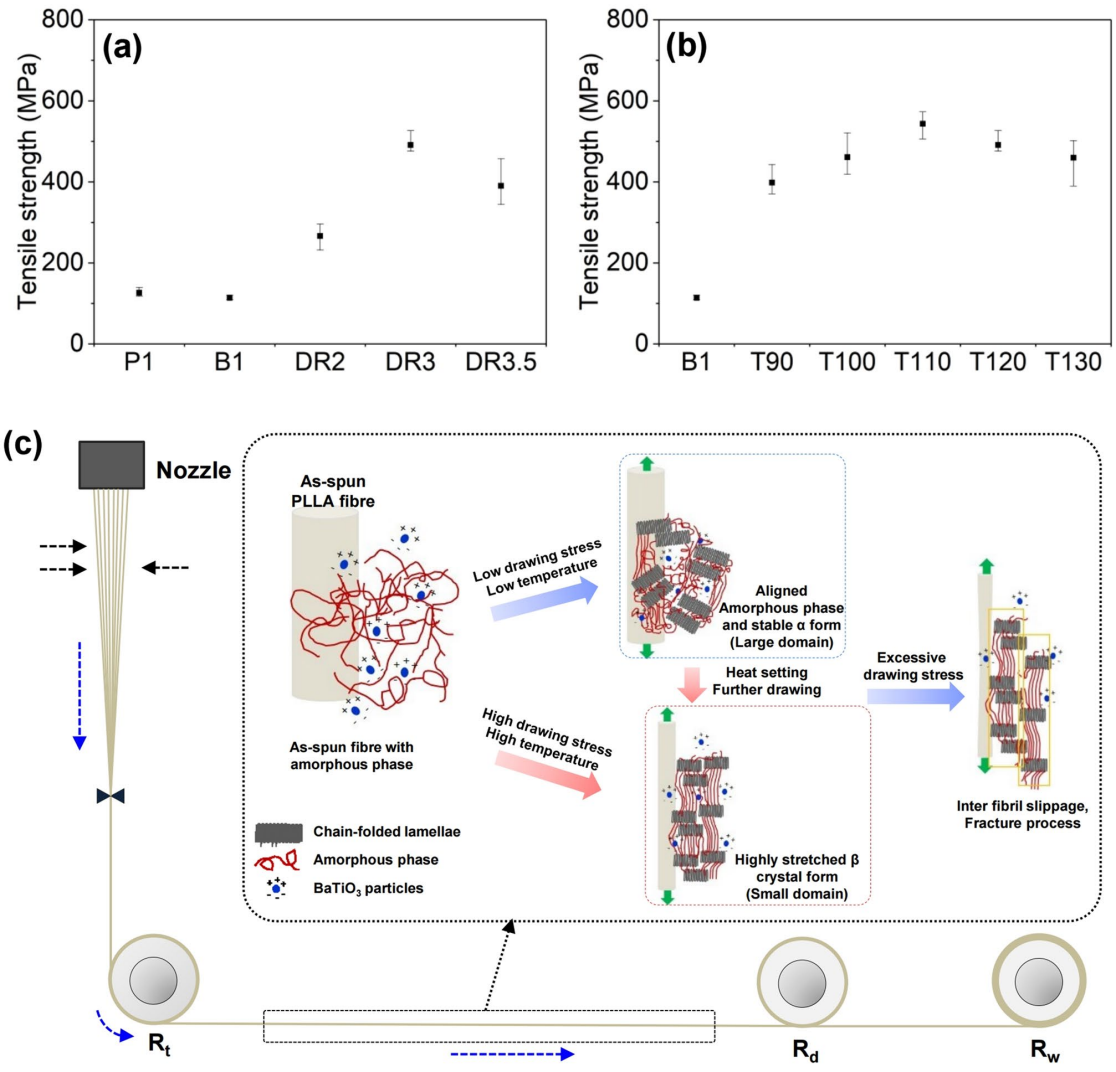
Tensile strengths of PLLA/BaTiO_3_ fibres treated with various (**a**) draw temperatures (T, °C) and (**b**) ratios (DR). (**c**) Mechanism of β-crystal formation in PLLA/BaTiO_3_ fibres during the drawing process.

The tensile strengths of PLLA/BaTiO_3_ fibres drawn at various draw ratios and temperatures are plotted in Fig. 5a,b. The tensile strength of as-spun P1 presented 113.7 MPa and it was slightly decreased by adding the BaTiO_3_ nanoparticles. As the draw ratio increased from 2 to 3, the tensile strength of the PLLA/BaTiO_3_ fibres was significantly increased, to a maximum of 491.2 MPa. In comparison, the tensile strength of the fibre obtained at a draw ratio of 3.5 was approximately 20% lower than that of DR3. In case of the drawing temperatures, the maximum tensile strength of the fibres obtained at a drawing temperature of 110 °C was 514.5 MPa, which was 3.7 times increase compared to that of the as-spun fibres. Above T110, the tensile strength decreased, which may have been due to the highly extended β-crystalline conformation in the PLLA/BaTiO_3_ fibres. In the melt-spinning process, the structural development of as-spun fibres was introduced by drawing process along the fibre axis (Fig. 5c). The amorphous phase of the as-spun fibre can be oriented and crystallized by a drawing stress in spin-line, and the comparatively stable α-crystalline phase be formed under a relatively lower drawing stress and temperature. Applying a greater drawing stress and temperature could transform the stable α-crystalline phase to the β form in the PLLA/BaTiO_3_ fibres^28–30^. However, the fracturing process in the PLLA/BaTiO_3_ fibres might occurs at higher draw ratios before fibre breakage^30,31^.

**Figure 6:**
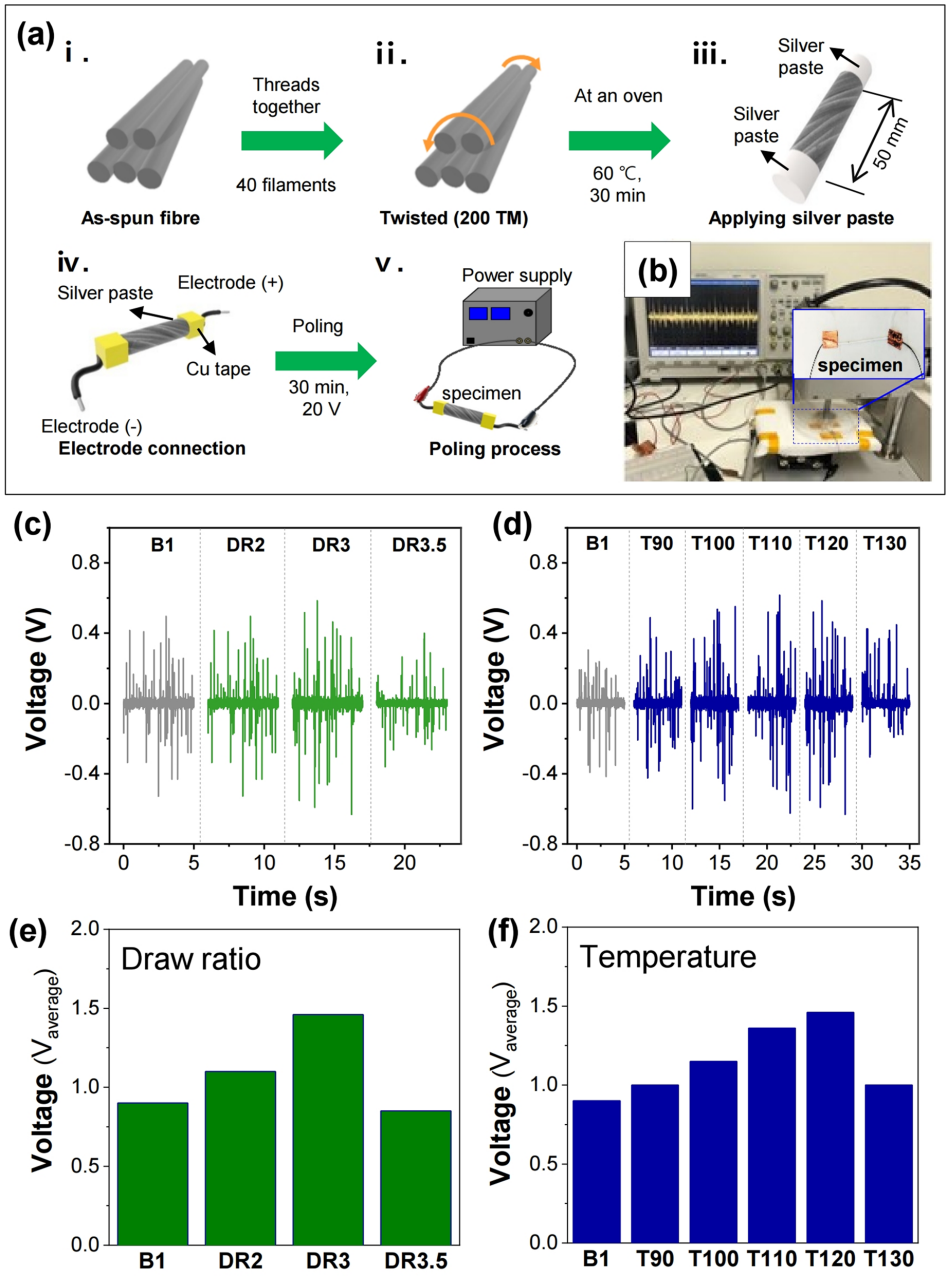
(**a**) Schematic of the specimen preparation and poling process for the piezoelectric test. (**b**) Piezoelectric test setup for the tapping method. Output voltages (V_pp_, peak to peak) of the PLLA/BaTiO_3_ filament yarn obtained at various (**c**) draw ratios (DR) and (**d**) temperatures (T, °C). Average output voltage tendency of the fibres produced with various (**e**) DR and (**f**) T.

The schematic of the specimen preparation and poling process for the piezoelectric test is presented in Fig. 6a. The actual experimental set-up is shown in Fig. 6b. The output voltages of PLLA/BaTiO_3_ fibres obtained at different draw ratios are plotted in Fig. 6c,e. The output voltage obtained increases to approximately 1.46 V up to a draw ratio of 3. Interestingly, when the draw ratio reaches 3.5, the output voltage diminishes to 850 mV. Figure 6d,f show the rise of the output voltage of the resulting fibres as the drawing temperature increased up to T120. The output voltage of the as-spun PLLA/BaTiO_3_ fibre is 900 mV, while those of the drawn fibres are 1, 1.15, 1.36, 1.46, and 1 V at drawing temperatures of 90, 100, 110, 120, and 130 °C, respectively. This increase in output voltage was attributed to the improved crystallinity and crystal transition of PLLA/BaTiO_3_ at higher drawing temperatures. These results were in good agreement with the DSC and XRD results shown in Figs. 3 and 4, respectively.

**Figure 7:**
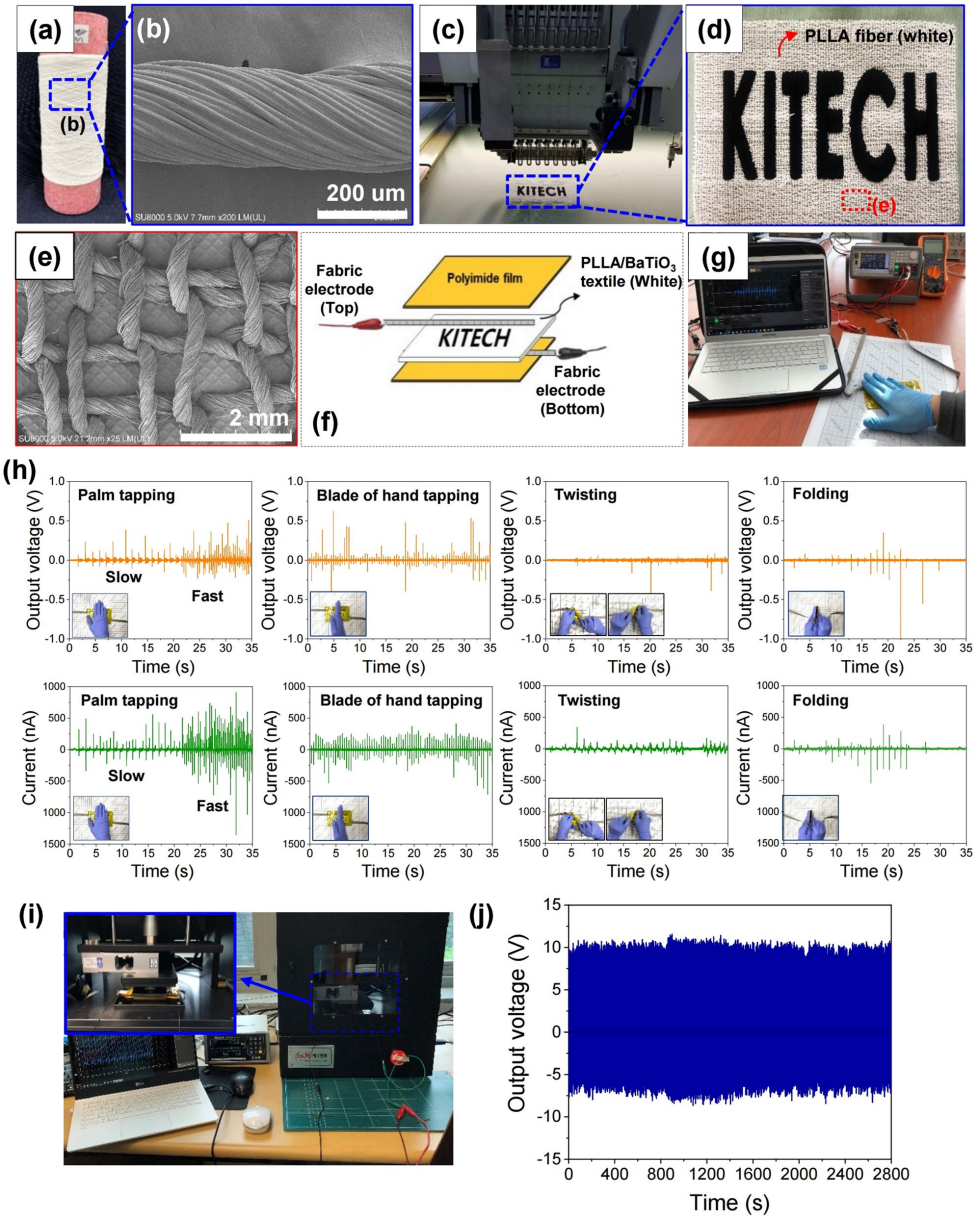
(**a**) Photograph of the twisted PLLA/BaTiO_3_ ply yarn. (**b**) FE-SEM image of PLLA/BaTiO_3_ ply yarn. Photograph of the (**c**) piezoelectric textile and the embroidery equipment, and (**d**) textile embroidered with PLLA/BaTiO_3_ fibres. (**e**) FE-SEM image of the embroidered structure of the PLLA/BaTiO_3_ fibre. (**f**) Schematic diagram of the device electrode. (**g**) Photograph of the piezoelectric test setup. (**h**) Output voltages and currents of the piezoelectric textile embroidered with PLLA/BaTiO_3_ fibres generated by various applied forces. (**i**) Stability test setup of the PLLA/BaTiO_3_ textile under a stress of 300 N by continuous tapping at 1 Hz for 2800 s. (**j**) Output voltages of the PLLA/BaTiO_3_ textile.

For practical applications, the PLLA/BaTiO_3_ piezoelectric fibres can be applied to fabrics or clothing. A piezoelectric textile made of the developed PLLA/BaTiO_3_ fibres (Fig. 7a,b) embroidering it in the fabric is shown in Fig. 7c,d. A top-down FE-SEM image of the embroidered PLLA/BaTiO_3_ ply yarn on the fabric is presented in Fig. 7e. The piezoelectric test setup in Fig. 7f–g was used to generate plots of output voltage and current vs. time with various mechanical deformations, such as tapping, twisting, and folding (Fig. 7h). The PLLA/BaTiO_3_ piezoelectric textile produced an output voltage up to 0.5 V with palm tapping and a maximum output voltage was 0.62 V with the tapping of the side of the hand. The highest output currents produced by the PLLA/BaTiO_3_ textile were observed when force was applied with the palm in tapping mode. With slow tapping, the piezoelectric textile produced output currents of up to 557 nA. The output current could be increased up to 911 nA with rapid tapping. An output current of approximately 411 nA was generated by tapping with the side of the hand over a smaller contact area. When the piezoelectric textile was twisted (Fig. 7h insets), the output voltage and current signal exhibited patterns that were distinct from those observed in tapping mode. Output voltages and currents of up to 0.55 V, and 345 nA were produced in twisting mode. In addition, we observed output currents as high as 383 nA in folding mode (Fig. 7h inset). Figure 7i shows the stability test setup by the application and release of pressure through a mechanical pressure machine at a frequency of 1 Hz and pressure force 300 N. The results showed that the electrical output voltages of the PLLA/BaTiO_3_ piezoelectric textile was relatively stable over an operation time of 2800 s (Fig. 7j).

## Discussion

In this study, we investigated piezoelectric enhancement of PLLA/BaTiO_3_ fibres by controlling the draw ratio and temperature of the pilot scale melt-spinning process. We confirmed that structural development of the PLLA/BaTiO_3_ fibres was accompanied by crystal-phase transitions that could be enhanced with higher draw ratios and temperatures. The b-phase transition of PLLA/BaTiO_3_ fibres exhibited as the draw ratio increased. However, it also could be undergo the fracture process with lamella slippage under an excessive draw ratio. In case of draw temperature, the crystal transition and crystallinity were increased up to 120 °C, but decreased again at higher temperature. This results is related to a decrease in tensile stress applied to the fibres as temperature increases. . The maximum output voltage was observed from the fibres obtained with a draw ratio of 3 and temperature of 120 °C. Under these conditions, we mass-produced PLLA/BaTiO_3_ fibres by melt-spinning and applied them to cotton fabric in embroidered form. The piezoelectric textile created from the developed PLLA/BaTiO_3_ fibres generated output currents of up to 911 nA in fast-tapping mode. Our flexible and stretchable PLLA/BaTiO_3_ fibre can be employed as an environmentally friendly sensor and may further the development of wearable electronics by the electronic textile industry. With these results, we confirmed the possibility of employing PLLA/BaTiO_3_ fibres as piezoelectric sensors in textile platform-based electronics. They could also be excellent substitutes for commercial piezoelectric materials based on heavy metals, because the PLLA/BaTiO_3_ fibres are environmentally friendly, flexible, and easy process to fabricate into textile devices.

## Method

### Materials

PLLA was purchased from Nature Works (6201D, USA), and BaTiO_3_ (99.9%) was obtained from Lumi-M Co, Ltd. (Seoul, Korea). The relative viscosity and melt flow rate of the PLLA were 3.1 and 15–30 g/10 min, respectively. The size distribution of BaTiO_3_ particles was 200–500 nm. Prior to the melt-spinning process, the polymer and BaTiO_3_ were dried at 80 °C for 12 h to bring the water content of the polymer chips to < 100 ppm.

### Melt spinning

The polymer (PLLA) matrix was reinforced with BaTiO_3_ nanoparticles. First, PLLA and BaTiO_3_ were mechanically mixed with a twin-screw compounding extruder. PLLA was introduced through the main feeder, and BaTiO_3_ powder was added through the side feeder. The materials were mixed by shear force with a roller speed of 700 rpm at 160 °C. The mixed molten polymer was then placed in a water bath to solidify at room temperature. Solidified polymeric strands of PLLA blended with BaTiO_3_ were then cut into chips for the melt-spinning process. In the melt-spinning process, PLLA/BaTiO_3_ polymer chips were first melted at 200 °C in the extruder. The molten polymer was then extruded through a spinneret with 24 extrusion holes, each with a diameter of 0.25 mm and a length-to-width (L/D) ratio of 2. The throughput rate and spinning temperature were fixed at 0.46 g/min and 190 °C, respectively. The distance between the spinneret and air quenching line was 4 m, and the take-up velocity was 1 km/min. The off-line post-drawing system consisted of four godet rollers (GR, GR1–GR4), and fibre drawing occurred between GR2 and GR3. During the post-drawing process, GR2 was fixed at 70 °C, which was above the glass transition temperature (*T*_g_) of the polymer. The draw ratio at GR3 varied between 2 and 3.5, which was below the breakage threshold of the fibres at 120 °C. The temperature of GR3 during fibre annealing was also varied from 90 to 130 °C.

### Characterization

The PLLA/BaTiO_3_ fibres obtained under various drawing conditions were then characterized. The surface and cross-sectional morphologies of the fibres were observed by field emission scanning electron microscopy (FE-SEM) (SU8010, Hitachi Co., Japan) at an accelerating voltage of 10 kV after sputter coating with gold. The dispersion of the BaTiO_3_ particles in the PLLA fibres (T120) was characterised using the BSE imaging mode at an acceleration voltage of 10 kV. The elements of the PLLA/ BaTiO_3_ fibres were confirmed by SEM–EDS at 20 kV. To determine the size distribution of the PLLA/BaTiO_3_ fibres, the cross-sectional diameters of at least 10 specimens were measured to report an average value. The thermal behaviour of the fibres was examined by DSC using a calorimeter (404 C, Netzsch, Germany) from 30 to 300 °C at a heating rate of 10 °C/min under N_2_. The crystal transformations and structural characteristics were determined by X-ray diffraction (XRD) analysis performed on a wide-angle X-ray diffractometer (Rigaku Denki Co., Japan) with Cu Kα1 radiation (λ = 1.54 Å) at a voltage of 40 kV and current of 40 mA. Data was collected at 2θ from 5° to 50°. 2D-WAXD patterns were recorded with an image plate detector at a camera length of 45 mm. The 2D-WAXD patterns were then reorganized into one-dimensional (1D) profiles for further analysis. The mechanical strength of the PLLA/BaTiO_3_ fibres was measured with a universal testing machine (Textechno, Favimat, Germany) according to ASTM standard D2256^32^. Each sample was prepared in the form of a single filament. The gauge length and crosshead speed for a single filament fibre were 20 mm and 20 mm/min, respectively. The test was conducted with at least 10 specimens, and the average value was recorded.

### Piezoelectric tests

The piezoelectric property was tested for the PLLA/BaTiO_3_ fibres at various drawing conditions (ratio, and temperature). 40 PLLA/BaTiO_3_ fibre filaments were twisted together by 200 twist per meter. To fix the twisted form, the filament yarn was treated at 60 °C for 30 min in an oven. Electrodes were affixed to both end of the PLLA/BaTiO_3_ fibres with silver paste with a distance of 50 mm between each electrodes. The filament yarns were poled for 30 min at a voltage of 20 V from a connected power supply (E36313A, Keysight Technologies, USA). The output voltage was measured using a DSO7054A oscilloscope (Agilent Technologies, USA) to characterise the piezoelectric properties of the PLLA/BaTiO_3_ fibres obtained under various draw conditions. Electrodes were affixed to both end of the PLLA/BaTiO_3_ fibres with silver paste, and Cu was used to form a contact between the electrode and the oscilloscope. We utilized a Berlincourt tester (AT Solution, Korea) and cyclic tapping method to apply a constant force of 2.9 N at 9 s^−1^. All voltage measurements were carried out at 295 K and 59% humidity.

The piezoelectric PLLA/BaTiO_3_ fibres were also employed in the textile. Fibres made with the pilot-scale melt-spinning process under optimized drawing conditions (draw ratio: 3; draw temperature: 120 °C) were gathered and twisted into a 300-denier ply yarn. The PLLA/BaTiO_3_ ply yarn was then embroidered into cotton fabric using an automatic embroidering machine. The embroidered PLLA/BaTiO_3_ dimensions in the fabric device were 98 × 50 mm. The fabric was poled with the corona poling apparatus by the same process used for the fibre bundles. Ni-coated conductive fabric was used as an electrode and attached to the top and bottom of the fabric device. The fabric device was covered with a polyimide film. The output current of the fabric device during application of various mechanical deformation was monitored with a DSOX4024A oscilloscope (Keysight Technologies, USA) and a B2902A source measurement unit (Keysight Technologies, USA). The stability test was performed by a mechanical pressure machine (SnM Tech, Korea) under a periodic external force of 300 N at 1 Hz. The data were measured and recorded by a source meter (Keysight, B2901A, USA), respectively.
